# Genetic Terminal Complement Deficiency in Israeli Bedouins With Kidney Failure

**DOI:** 10.1016/j.ekir.2025.01.019

**Published:** 2025-01-20

**Authors:** Guy Chowers, Dror Ben-Ruby, Danit Atias-Varon, Omer Shlomovitz, Keren Slabodnik-Kaner, Maayan Kagan, Shany Avayou, Elvira Romanjuk, Boris Rogachev, Yosef S. Haviv, Ohad S. Birk, Noam Hadar, Younes Bathish, Iris Barshack, Alexander Volkov, Camila Avivi, Anna Pavlovsky, Orly Haskin, Amos J. Simon, Efrat Glick-Saar, Alina Ostrovsky, Mawada Assi, Ruth Schreiber, Dana Levin, Yoram Yagil, Mohammad Awawdeh, Karl Skorecki, Dan Dominissini, Alla Shnaider, Asaf Vivante

**Affiliations:** 1 Department of Pediatrics B, Edmond and Lily Safra Children's Hospital, Sheba Medical Center, Tel-Hashomer, Ramat-Gan, Israel; 2 Faculty of Medical and Health Sciences, Tel-Aviv University, Tel-Aviv, Israel; 3 Genetic Kidney Diseases Research Laboratory, Sheba Medical Center, Tel-Hashomer, Ramat-Gan, Israel; 4 Department of Nephrology, Soroka University Medical Centre, Beer-Sheva, Israel; 5 Faculty of Health Sciences, Ben Gurion University, Beer-Sheva, Israel; 6 Genetics Institute at Soroka Medical Center, Beer-Sheva, Israel; 7 Ziv Medical Center, Safed, Israel; 8 The Azrieli Faculty of Medicine, Bar-Ilan University, Safed, Israel; 9 Institute of Pathology, Sheba Medical Center, Tel-Hashomer, Ramat-Gan, Israel; 10 Institute of Nephrology, Schneider Children’s Medical Center of Israel, Petach Tikva, Israel; 11 Hemato-Immunology Unit, Hematology Laboratory, Sheba Medical Center, Tel-Hashomer, Ramat-Gan, Israel; 12 Sheba Cancer Research Centre, Sheba Medical Center, Tel-Hashomer, Ramat-Gan, Israel; 13 Wohl Center for Translational Medicine, Sheba Medical Center, Tel-Hashomer, Ramat-Gan, Israel; 14 Pediatric Nephrology Clinic, Soroka University Medical Center, Beer-Sheva, Israel; 15 A.P.C Health, Ramat-Gan, Israel; 16 Department of Nephrology and Hypertension, Barzilai University Medical Center, Ashkelon, Israel; 17 Hemodialysis Unit, Department of Internal Medicine, Holy Family Hospital, Nazareth, Israel; 18 Rappaport Faculty of Medicine and Research Institute, Technion-Israel Institute of Technology, Haifa, Israel; 19 Rambam Health Care Campus, Haifa, Israel; 20 Pediatric Nephrology Unit, Edmond and Lily Safra Children's Hospital, Sheba Medical Center, Tel-Hashomer, Ramat-Gan, Israel

**Keywords:** chronic kidney diseases, complement, ethnic minorities, exome sequencing, kidney failure, terminal complement complex

## Introduction

The prevalence of chronic kidney disease (CKD) is particularly high among Israeli non-Jewish populations,S1^,^S2 often attributed to genetic kidney diseases.^1^ We established the Israeli Kidney Failure Genetic Cohort to reveal the genetic causes of kidney failure in adults across diverse ethnicities.^1^ Here, we studied the Israeli Bedouins, a seminomadic, Arabic-speaking minority. The Bedouin social structure is based on nuclear families integrated into tribes with high endogamy and consanguinity rates nearing 45%.^2^^,^S3^,^S4 The Bedouin population primarily resides in North Africa, the Sinai and Arabian Peninsulas, as well as in Israel's southern Negev desert and Galilee region. In this pilot study, we aimed to uncover the genetic landscape of kidney failure in Israeli Bedouins by focusing on identifying high-penetrance monogenic causes using exome sequencing (ES).

## Results

Clinical characteristics of the study population (*N* = 81) are presented in Supplementary Table S1. Using ES, we identified genetic causes in 10 participants (12%), including 6 distinct monogenic disorders (Table 1).^3^^,^S5-S10 The mean age at kidney failure diagnosis, along with rates of parental consanguinity and family history of kidney failure were similar for patients with genetically driven kidney failure compared to patients with negative ES. Pathogenic *COL4A3* variants were detected in 5 participants, making *COL4A3* variants the most common molecular genetic diagnosis in our cohort (Supplementary Figure S1). Genetic diagnosis was often discordant with the initial clinical diagnosis and influenced subsequent clinical management (Supplementary Table S2).

**Table 1:** Clinical data and post–exome sequencing molecular-genetic diagnoses in 13 Bedouin individuals in whom diagnostic variants were identified

Patient	Pre–exome sequencing clinical diagnosis	FHx	Post–exome sequencing diagnosis	Gene	Annotation	Zygosity	CADD score^a^	AF gnomAD^b^	AF in matched controls (*n* = 399)^c^	ACMG^d^	ACMG criteria^d^
Monogenic kidney diseases											
21–759	Male with ADPKD, hearing loss; kidney failure at age of 54 yrs	Yes	Type 4 collagen related CKD (Alport syndrome spectrum)	*COL4A3*	c.361G>A, p.Gly121Ser	Hom	25.2	0.0009%	1∖798 0.13%	LP	PM2 (Supp), PM1, PP3 (Mod), PM3 (Supp)
21–1209	Male with diabetic nephropathy; kidney failure at age of 64 yrs	Yes	Type 4 collagen related CKD (Alport syndrome spectrum)	*COL4A3*	c.361G>A p.Gly121Ser	Het	25.2	0.0009%	1∖798 0.13%	LP	PM2 (Supp), PM1, PP3 (Mod), PM3 (Supp)
21–990	Male with nephropathy of unknown cause; kidney failure at age of 35 yrs	Yes	Type 4 collagen related CKD (Alport syndrome spectrum)	*COL4A3*	c.2275G>C, p.Gly759Arg	Het	39	0.0001%	3∖798 0.38%	LP	PP3 (Mod), PM1, PM2 (Supp), PS1 (Supp),
21–1614	Female with nephropathy of unknown cause; kidney failure at age of 54 yrs	No	Type 4 collagen related CKD (Alport syndrome spectrum)	*COL4A3*	c.2275G>C, p.Gly759Arg	Het	39	0.0001%	3∖798 0.38%	LP	PP3 (Mod), PM1, PM2 (Supp), PS1 (Supp),
21–715	Female with diabetic nephropathy; kidney failure at age of 73 yrs	No	Type 4 collagen related CKD (Alport syndrome spectrum)	*COL4A3*	c.3829G>T p.Gly1277Cys	Het	25.8	0.0%	1∖798 0.13%	LP	PP3 (Mod), PM2 (Supp), PM5, PP2
21–755	Female with GSD type 1b, hearing loss, and intellectual disability; kidney failure at age of 20 yrs; parental consanguinity	No	Glycogen storage disease type 1b	*SLC37A*	c.1042_1043 delCT p.Leu348fs	Hom	35	0.018%	0/798 0%	P	PVS1, PM2 (Supp), PS4
21–1028	Female with FSGS, hypothyroidism; kidney failure at age of 20 yrs	No	Papillorenal syndrome	*PAX2*	c.76dupG p.Val26fs	Het	37	0.0005%	0/798 0%	P	PVS1, PM2 (Supp), PS2
21–1064	Male with nephropathy of unknown cause, heart failure; kidney failure at age of 20 yrs; parental consanguinity	No	Nephronophthisis 4	*NPHP4*	chr1: g.5964667_ 6008322del	Hom	N/A^e^	N/A	N/A	P	2E, 4L
21–1210	Female with hemolytic uremic syndrome; kidney failure at age of 52 yrs	Yes	Atypical HUS	*CFH*	c.3493+5G>A	Het	22.6	0.0%	0/798 0%	LP^f^	PP3, PS1 (Mod), PM2 (Supp), PS4 (Mod), PP4
21–1311	Male with ADPKD; kidney failure at age of 48 yrs	Yes	ADPKD	*PKD2*	c.1390C>T p.Arg464∗	Het	37	0.0%	0/798 0%	P	PVS1, PM2 (Supp), PS4
Other monogenic diseases (terminal complement)											
21–179	Male with IgA nephropathy; kidney failure at age of 13 yrs; parental consanguinity	No	Terminal complement deficiency	*C8A*	c.630C>A p.Tyr210∗	Hom	41	0.0008%	11/798 1.38%	P	PVS1, PM2 (Supp), PS3
21–1016	Male with IgA nephropathy; kidney failure at age of 13 yrs; parental consanguinity	No	Terminal complement deficiency	*C8A*	c.630C>A p.Tyr210∗	Hom	41	0.0008%	11/798 1.38%	P	PVS1, PM2 (Supp), PS3
21–753	Male with diabetic nephropathy; kidney failure at age of 32 yrs; parental consanguinity	No	Terminal complement deficiency	*C8B*	c.1282C>T p.Arg428∗	Hom	39	0.13%	2/798 0.25%	P	PVS1, PM2 (Supp), PS3

ACMG, American College of Medical Genetics classification; ADPKD, autosomal dominant polycystic kidney disease; AF, allele frequency; B, benign; CADD score, combined annotation dependent depletion score; CAKUT, congenital anomalies of the kidney and urinary tract; CKD, chronic kidney disease; DM, diabetes mellitus; FHx, family history of kidney disease; FSGS, focal segmental glomerulosclerosis; gnomAD, genome aggregation database; GSD, glycogen storage disease; Het, heterozygous; Hom, homozygous; HUS, hemolytic uremic syndrome; LB, likely benign; LP, likely pathogenic; Mod, moderate; P, pathogenic; Supp, supporting; VUS, variant of unknown significance.

Only 1 patient had a prior molecular diagnosis (glycogen storage disease type 1b) and 2 participants had a clinical phenotype-based suspicion of ADPKD; One participant was confirmed to have a pathogenic *PKD2* variant, the other participant had a homozygous *COL4A3* variant classified as likely pathogenic. *COL4A* pathogenic variants have recently been reported as a cause of cystic kidney disease, which can mimic ADPKD.^3^^,^S5^,^S6 A manual inspection of *PKD1* and *PKD2* to verify sequencing adequacy was conducted, confirming 98% and 100% of regions covered at >20x depth, respectively, thus minimizing the likelihood of undetected pathogenic variants in these genes.

a *In silico* prediction tool for scoring the deleteriousness of variants, higher values of CADD score predict a more severe effect on a logarithmic scale.S7

b According to the gnomAD version 4.1.0.S8

c In-house controls include 399 Bedouins from the Negev region in Israel.

d ACMG: according to the ACMG classification, a variant can be classified as P, LP, VUS, LB; or B. All variant classified with the https://franklin.genoox.com platform. The level of evidence was assigned based on the ACMG guidelines and the updated ClinGen SVI recommendations. Any adjustments made to these default classifications are indicated in brackets.

e Neither the exact deletion nor a similar deletion with > 50% overlap were found using CADD-SV tool.S7

f Application of the PS4 criterion based on previous reports of this variant in two affected cases and absence in controls.S9 PP3 and PS1 (Mod) used based on ClinGen SVI guidelines for splice variants.S10

### Terminal Complement Pathogenic Variants Among Bedouins With Kidney Failure

**Figure 1:**
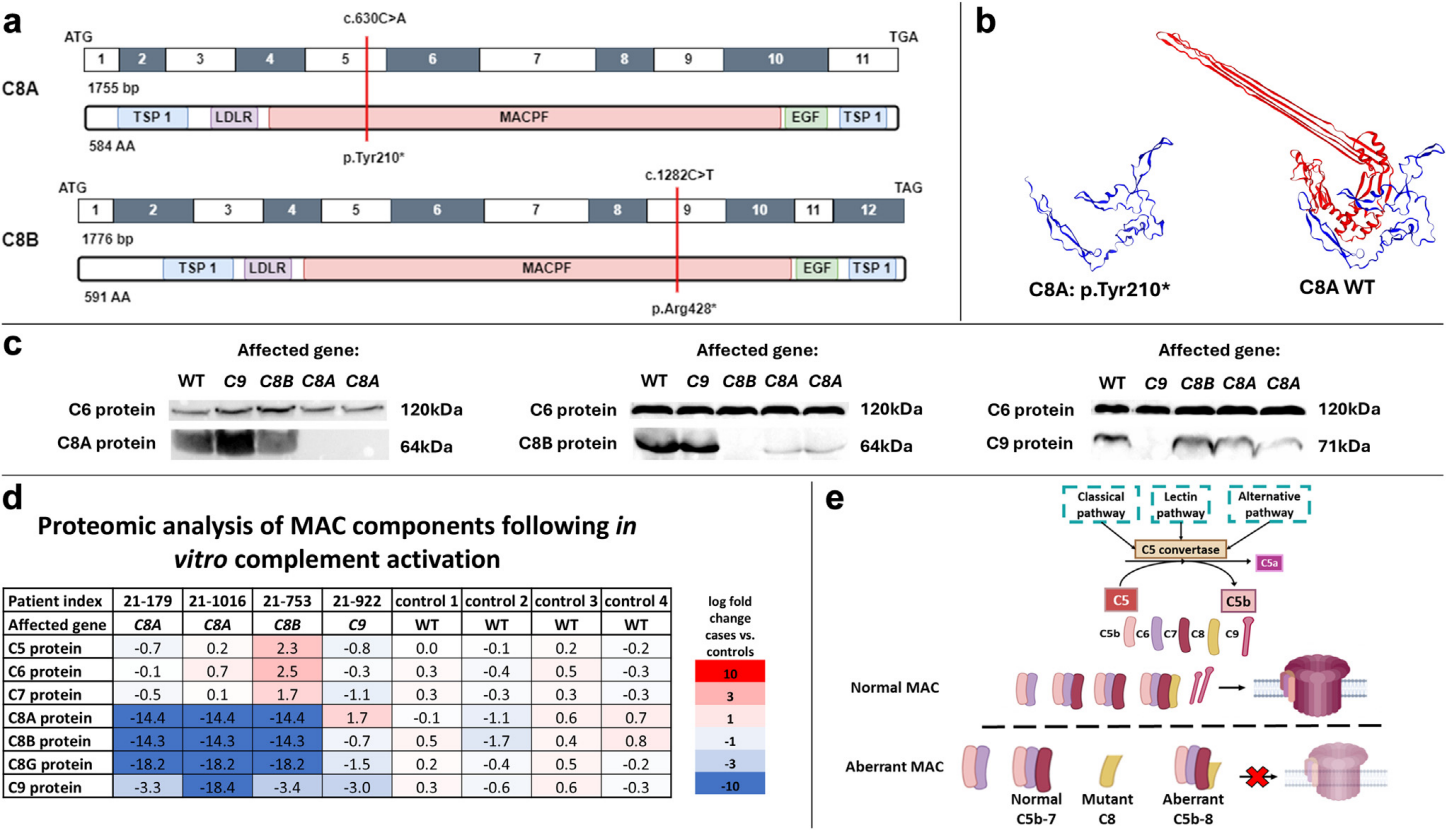
Identification of variants in *C8A* and *C8B* in 3 Israeli Bedouin individuals with kidney failure. (a) Exon structure of human *C8A* and *C8B* cDNA and domain structure of complement factor 8 alpha and beta chains (C8A and C8B). Nucleotide and protein changes are highlighted by red lines. Both variants were located in the MACPF domain. This domain is crucial for target membrane insertion and the formation of a transmembrane channel that disrupts the cell, causing lysis. ATG and TGA/TAG stop codons are indicated. (b) 3D model of C8A with and without the truncating variant. The model was generated the SWISS-MODEL.S16 The N terminus upstream of the early stop codon at residue 210 is shown in blue. The C terminus, downstream of the early stop codon, is shown in red. (c) Western blot of serum from patients with deficits in terminal complement proteins. When probing the C8B chain, in addition to the anticipated protein absence in the case of the *C8B* variant, the 2 cases with the *C8A* variant showed reduced protein levels. The label in each lane indicates the affected gene in each patient, including 1 healthy, unrelated individual as a control (labeled WT). C6 was used as the loading control. (d) Proteomic analysis of the MAC components showing aberrant MAC formation. Complexes were analyzed following *in vitro* complement activation and pull-down of C6 containing complexes using co-IP. Each sample was compared to the average protein levels of controls. Whereas complete C5b-9 MAC were formed in controls, very low levels of C8 and C9 were detected in the cases. (e) Normal complement cascade and suggested pathological mechanisms leading to kidney disease. Abnormal C8 or C9 proteins result in the inability of C9 monomers to connect to the forming MAC and the absence of intact membranes penetrating pores. The resulting partial MAC, lacking C9 and some C8 chains, may lead to complement deposition in the glomeruli, either by itself as protein aggregates or as a part of immune complexes. co-IP, coimmunoprecipitation; EGF, epidermal growth factor like domain; LDLR, low density lipoprotein receptor class A domain; MAC, membrane attack complex; MACPF, membrane attack complex/perforin domain; TSP1, thrombospondin 1 domain.

Three unrelated patients were found to harbor nonsense variants in 2 terminal complement genes, 1 patient with *C8B* variant and 2 patients with *C8A* variant (Supplementary Figure S1, Table 1^3^^,^S5-S10). These genes translate to the α and β chains of complement factor 8, which comprise the membrane attack complex (MAC)—together with C5-9—the final common pathway of the complement cascade. These 2 variants have been previously reported in the context of C8 deficiency, which causes susceptibility to encapsulated infections.^4^^,^^5^ Both truncating variants are located within the MAC/perforin domain (Figure 1a and b).

Patient 21-179 with a *C8A* pathogenic variant developed kidney failure at the age of 13 years. A renal biopsy revealed mesangial IgA deposits (Supplementary Figure S2, Table 1^3^^,^S5-S10). The patient experienced rapid progression despite treatment and eventually required dialysis. Subsequently, he underwent 2 kidney transplantations, which were lost because of acute rejection attributed to thrombotic microangiopathy and recurrence of IgA and C3 deposits, respectively. Similarly, patient 21-1016 presented at age 9 with elevated creatinine levels and a kidney histopathological pattern of IgA nephropathy. He progressed to kidney failure by the age of 13 years and required 3 transplants over the next 20 years because of recurrent rejection. Patient 21-753 with *C8B* variant presented with type 2 diabetes mellitus and progressive proteinuria, leading to kidney failure at age of 32 years. Unfortunately, renal biopsy was not performed. None of the patients had a history of infection before or after the onset of kidney disease.

C8 deficiency has not previously been associated with kidney disease. However, the *C8A* variant was significantly enriched in our cohort compared to ethnically matched controls. The homozygous genotype was enriched among cases compared with controls (*P* = 0.04, Fisher exact test). The minor allele frequency was higher in cases than in controls (3.7% vs. 1.38%, *P* = 0.04). This enrichment was driven by a higher proportion of homozygotes (2/81 vs. 0/399, *P* = 0.002) rather than by differences in carrier rates (2/81 vs. 11/399, *P* = 0.9). This enrichment led us to screen for variants in the terminal complement genes among 1300 Israeli patients with kidney failure. Consequently, we identified an Arab-Israeli patient with a likely pathogenic homozygous variant in *C9* (c.355T>G, p.Cys119Gly). Unfortunately, this patient did not undergo renal biopsy.

### Terminal Complement Variants Result in Aberrant MAC Formation

To study the association between terminal complement variants and kidney injury, we examined the deleterious effects of these variants. We measured the presence of each component of the complement cascade in the patients’ serum and total hemolytic activity using the CH50 assay (Supplementary Table S3). Reduced levels of C8 and C9 proteins were observed in 4 participants harboring *C8A, C8B*, or *C9* pathogenic variants, respectively. Accordingly, all 4 cases had low CH50 levels, indicating an impaired ability to form a functional MAC. The other components of the complement system were not remarkably altered (Supplementary Table S3). Western blot analysis showed the absence of the corresponding proteins in all cases, consistent with the identified pathogenic variants (Figure 1c).

We hypothesized that the formation of aberrant MACs due to the inability to connect C9 monomers to the initial C5b-7 complex, may contribute to kidney disease. To detect these partial complexes following complement activation, we performed coimmunoprecipitation, followed by mass spectrometry (Figure 1d and e). Mass spectrometry revealed that in patient sera, incomplete MACs were formed and composed only of C5b-7 components (or C5b-8 in the case of the C9 variant). This suggests that following complement activation, aberrant MACs are formed in affected patients, which may subsequently result in uncleared glomerular depositions.

## Discussion

In this nationwide study, exome sequencing of 81 Israeli Bedouins with kidney failure identified diagnostic variants in 12% of cases that were often clinically undiagnosed. In addition, we identified a cluster of monogenic terminal-complement deficiencies, suggesting a potential novel link to CKD.

Our findings provide several insights. First, the incidence of monogenic kidney disease among the Bedouin population is higher than previously reported in heterogeneous populations of European ancestry.^6^ Given the underrepresentation of Bedouins in genetic databases, our findings underscore the need for more diverse international study cohorts. Second, the low rate of previous genetic diagnoses and poor concordance with pre-exome diagnoses highlights the genetic and phenotypic heterogeneity of CKD. Furthermore, in contrast to previous studies suggesting that *PKD1* and *PKD2* are the most common genetic causes of kidney failure,^6^^,^^7^ our findings indicate that among Bedouins, *COL4A3* variants are the predominant genetic etiology. Consistent with previous studies,^1^^,^S11 our findings suggest that genetic CKD is characterized by a population-specific genetic landscape.

Finally, our results support a possible emerging role of aberrant terminal complement activity in CKD. Strikingly, 2 patients with *C8A* variants were diagnosed with kidney failure due to IgA nephropathy, requiring kidney replacement therapy by the age of 13 years. This extremely rare childhood phenotype suggests a potential contributory role of *C8A* pathogenic variants. Whereas research on complement dysregulation in kidney disease has focused on early cascade components, the role of terminal complement, particularly its link to CKD and IgA nephropathy, remains less understood, with associations to renal disease being inconsistent.^8^^,^^9^^,^S12 A French cohort study reported noninfectious or autoimmune diseases, including kidney disease, in 16 of 154 patients with terminal complement deficiencies,^4^ whereas in a UK cohort of 40 such patients kidney involvement was not reported.^5^ Notably, C8 and C9 knockout murine models have not been assessed for kidney injury.S13^,^S14

Our results indicated reduced functional complement activity in all 4 patients with terminal complement variants. We hypothesize that this deficiency contributes to kidney disease via the formation of incomplete MAC deposits in the glomeruli, either independently or within immune complexes. It is plausible that such deposition may require a “second hit” trigger, like an infection, to activate the complement system, potentially exacerbating existing conditions.^8^^,^^9^ Alternatively, impaired MAC formation might lead to overactivation of early complement components, a mechanism linked previously to C3 glomerulopathy and membranoproliferative glomerulonephritis,^8^ or defective clearance of apoptotic cells, possibly triggering autoimmunity.S15 In summary, the high prevalence of terminal complement deficiencies in our cohort suggests a possible role in kidney disease. These deficiencies may act as primary causes of kidney disease or as exacerbating factors in various kidney pathologies, possibly mediated by the formation of aberrant MACs. These insights may have clinical implications, both for precise genetic diagnosis and possible employment of available complement targeting therapies. Further studies with larger and more diverse cohorts are needed to better understand the underlying molecular mechanisms.

## Disclosure

The authors declare that they have nothing to disclose.

## Patient Consent

Written informed consent was obtained from all participants.

## Supplementary Material

Supplementary File (PDF) **Supplementary Methods. Supplementary References. Figure S1.** Diagnostic yield of exome sequencing in the Israeli Bedouin kidney failure population. **Figure S2.** Histopathology of kidney biopsy obtained from 179-21 (C8A truncating variant) showing IgA nephropathy. **Table S1.** Clinical characteristics and pre-exome sequencing diagnosis of 81 Bedouin persons with kidney failure. **Table S2.** Effect of post-ES molecular-genetic diagnoses on clinical management. **Table S3.** Total serum complement activity (CH50), complement components and immunoglobulins level in patients with kidney failure and pathogenic variants in terminal complement genes.

## Footnotes

Supplementary File (PDF)

**Supplementary Methods.**

**Supplementary References.**
**Figure S1.** Diagnostic yield of exome sequencing in the Israeli Bedouin kidney failure population. **Figure S2.** Histopathology of kidney biopsy obtained from 179-21 (C8A truncating variant) showing IgA nephropathy. **Table S1.** Clinical characteristics and pre-exome sequencing diagnosis of 81 Bedouin persons with kidney failure. **Table S2.** Effect of post-ES molecular-genetic diagnoses on clinical management. **Table S3.** Total serum complement activity (CH50), complement components and immunoglobulins level in patients with kidney failure and pathogenic variants in terminal complement genes.
