# Supplementary material for: Genetic Terminal Complement Deficiency in Israeli Bedouins With Kidney Failure
Source: Kidney Int Rep. 2025 Jan 20;10(4):1274–8. doi: 10.1016/j.ekir.2025.01.019 (PMC12034871; doi:10.1016/j.ekir.2025.01.019)
Supplement: Supplementary File (PDF) — Supplementary Methods. Supplementary References. Figure S1. Diagnostic yield of exome sequencing in the Israeli Bedouin kidney failure population. Figure S2. Histopathology of kidney biopsy obtained from 179-21 (C8A truncating variant) showing IgA nephropathy. Table S1. Clinical characteristics and pre-exome sequencing diagnosis of 81 Bedouin persons with kidney failure. Table S2. Effect of post-ES molecular-genetic diagnoses on clinical management. Table S3. Total serum complement activity (CH50), complement components and immunoglobulins level in patients with kidney failure and pathogenic variants in terminal complement genes. [file mmc1.pdf]

Genetic analysis among Israeli Bedouins with kidney failure reveals terminal complement pathogenic variants

Guy Chowers M.D.[1,2,3*], Dror Ben-Ruby M.Sc[2,3*], Danit Atias-Varon Ph.D.[1,3] ,Omer Shlomovitz M.D.[1,2,3], Keren Slabodnik-Kaner M.Sc[1,3], Maayan Kagan M.D.[1,2,3], Shany Avayou M.D.[3], Elvira Romanjuk M.D.[4,5], Boris Rogachev M.D.[4,5], Yosef S Haviv M.D.[4,5], Ohad S. Birk M.D., Ph.D.[5,6], Noam Hadar Ph.D.[5], Younes Bathish M.D.[7,8], Iris Barshack M.D.[2,9], Alexander Volkov M.D.[2,9], Camila Avivi Ph.D.[9], Anna Pavlovsky Ph.D.[9], Orly Haskin M.D.[2,10], Amos J. Simon Ph.D.[11,12], Efrat Glick-Saar Ph.D [12,13], Alina Ostrovsky M.Sc [11], Mawada Assi B.Sc[11], Ruth Schreiber M.D.[5,14], Dana Levin M.D.[3], Yoram Yagil M.D.[5,15,16], Mohammad Awawdeh M.D.[8,17], Karl Skorecki M.D [8,18,19], Dan Dominissini Ph.D.[2,11,12,13], Alla Shnaider M.D.[4,5] and Asaf Vivante M.D., Ph.D.[1,2,3,20].

Supplementary materials

Supplementary material table of contents

Supplementary methods: page 3

Supplementary table 1: page 9

Supplementary table 2: page 11

Supplementary table 3: page 14

Supplementary figure 1: page 15

Supplementary figure 2: page 16

Supplementary references: page 17

Supplementary methods

Study population

We conducted a national multicenter prospective study involving all Israeli dialysis units and established the Israeli Kidney Failure Genetic Cohort as outlined previously[1]. Utilizing data from the Israeli Dialysis Registry, we identified and included a substantial cohort of adult Israeli Bedouin patients (81 individuals) undergoing kidney replacement therapy, mainly dialysis treated. The estimated prevalence of dialysis treated Bedouins in Israel is about 250 individuals based on national registries.

Following obtaining informed consent, from December 2021 to August 2023, we prospectively gathered clinical data, pedigree information, and blood samples from individuals attending dialysis or post-transplant clinics using a standardized questionnaire[1]. Approval for human subject research was granted by the Institutional Review Boards of Sheba Medical Center and the Israeli Ministry of Health, as well as by the ethics review boards of participating centers. Participant recruitment for this study was conducted under the auspices of an ongoing national initiative approved by the Ministry of Health's Institutional Review Board in adherence with the principles outlined in the Declaration of Helsinki.

Clinical assessment and diagnosis

A single or multiple primary clinical diagnoses were recorded for each participant based on their medical records and their primary clinical nephrology assessment. In addition, all participants underwent a clinical interview at the time of study recruitment, which included a comprehensive review of their medical files, imaging studies and kidney biopsy information when available. The primary kidney clinical diagnoses were classified as either diabetic nephropathy, hypertensive

nephropathy, glomerulopathy, polycystic kidney disease, congenital anomalies of the kidney and urinary tract (CAKUT), tubulo-interstitial disease, nephrolithiasis, nephropathy of unknown cause and other diagnoses. Furthermore, information regarding the medical history, family history, timing of kidney failure, status of consanguinity, and extra-renal manifestations were obtained (**Table S1**).

Exome Sequencing

DNA was isolated from PBMCs using Wizard Genomic DNA Purification Kit (Promega) according to the manufacturer's instructions. Proband exome sequencing was performed on genomic DNA for all affected individuals using an IDT xGeb Exome Hyb Panel V2 and Illumina NoveSeq6000 sequencing technology. For each sample, paired end-reads (2×100bp) were obtained, processed, and mapped to the genome. The BWA-MEM algorithm Version 0.7.17-r1188 was used to align the sequence reads to the human reference genome (hg37). The HaplotypeCaller algorithm of GATK V.4.4.0.0 was applied for variant calling, as recommended in the best practice pipeline[S17].

Variant interpretation

Exome sequencing data was filtered using the Franklin Platform (Genoox, Tel-Aviv, Israel, available at https://franklin.genoox.com). Exome data were interpreted following the American College of Medical Genetics and Genomics (ACMG) guidelines[S18], incorporating the latest recommendations from the Clinical Genome Resource (ClinGen) Sequence Variant Interpretation (SVI) Working Group (available at: https://clinicalgenome.org/working-groups/sequence-variant-interpretation). We classified variants as pathogenic, likely pathogenic, variants of unknown significance (VUS), likely benign or benign. ACMG standards and guidelines recommend 28

criteria for variant pathogenicity classification. The Genoox Classification Engine ran 17 of them automatically. The artificial intelligence-based variant classification engine automates rules PVS1, PS1, PM1, PM2, PM4, PM5, PP2, PP3, PP5, BA1, BS1, BS2, BP1, BP3, BP4, BP6, and BP7[S19]. The remaining rules cannot be automated, as they require clinical information specific to the patient genotype, e.g., familial data, de novo evidence (PS2, PM6), segregation data (PP1, BS4), and/or allelic data (PM3, BP2). Therefore, the remaining criteria were manually classified. We evaluated all variants reported pathogenic or likely pathogenic by Clinvar and by the Genoox Classification Engine, all rare (MAF < 1%) homozygous variants and all rare (MAF < 1%) heterozygous variants found in our in-house CKD panel as previously published[1].

CNV were also called using the Franklin platform, utilizing the AI algorithm “Rainbow”. developed by Genoox. Rainbow generates a model for copy number prediction by using a learning set of over 30 sequenced samples from the cohort. It calculates the predicted coverage of each exon based on more than 50 unique predictors, including various exons or regions, ideally from different chromosomes, whose coverage is statistically correlated with that of the exon (https://help.genoox.com/en/articles/5365431-cnv-detection-byfranklin). Rainbow then predicts the exon copy number and assigns a prediction score.

Complement studies

Protein Structure Modeling: 3D structure of wild type and truncated complement proteins was predicted using the SWISS-MODEL software[S16].

Complement Assays: Complement measurements were performed using standard clinically approved techniques. Total complement function was measured using CH50 ELISA assay (MicroVue CH50 Eq EIA, Quidel). Specific complement components and immunoglobulins were

detected by commercially available kits using common techniques such as radial immunodiffusion (RID, The Binding Site) or nephelometry (BNII, Siemens).

Western Blot: 8% Bis-Acrylamide separation gel was prepared using 5ml Tris 1.5M pH 8.8, 200µl SDS 10%, 4ml Bis-Acrylamide 40%, 100µl APS 20%, 10µl TEMED, and DDW to a total of 20ml. 4% Bis-Acrylamide stacking gel was prepared using 2.5ml Tris 0.5M pH 6.8, 100µl SDS 10%, 1ml Bis-Acrylamide 40%, 50µl APS 20%, 10µl TEMED and DDW to a total of 10ml. Samples were reduced and denatured by Laemmli SDS-Sample Buffer (Bioprep) for 5 min in 95°C. The samples were then separated by electrophoresis and transferred to nitrocellulose membrane. The membrane was blocked with 8% skim milk in TBST for 30 min in room temperature. Primary antibody (C6-Invitrogen, PA572500; C7- Invitrogen, PA5-103761; C8A- Abcam, ab273626; C8B- Novus Bio- NBP185990; C9- Santa Cruz, SC69761) was let to hybridize in 5% BSA solution in TBST (overnight, 4°C). Secondary antibodies (Jackson Immunoresearch 115035062 and 111035144) were incubated in 5% skim milk TBST solution, and color was developed using ECL (Cyanagen). Images were captured using Bio-Rad ChemiDoc MP imaging system, and analyzed using ImageLab software (Bio-Rad).

Proteomic analysis of membrane attack complexes

Co-Immunoprecipitation (Co-IP): To isolate membrane attack complexes, we *in vitro* activated patients' sera, followed by C6 targeted Co-IP. 200µl serum was activated using 2µl heat aggregated gamma globulin (Quidel, A114) and incubated at 37°C for 90 min. 50µl of Dynabeads Protein G (Invitrogen, 10003D) were washed using 200µl 0.02% PBS Tween. Then, 4.5µg of anti-C6 antibody (Invitrogen, PA572500) was conjugated to the beads (20 min incubation, room temperature). The activated serum was then added to the antibody conjugated beads, and incubated

for 120 min, room temperature. The beads were washed with fresh PBS and were taken fresh for further processing for mass spectrometry.

On-Bead Digestion: The beads were resuspended in 100 µl of Elution Buffer I (2M UREA/50 mM Tris-HCl pH 7.5/1 mM DTT), and proteins were digested by the addition of 0.5 µg of sequencing-grade trypsin (Promega). After 1 hour of incubation at room temperature with shaking, the beads were separated on a magnetic rack, and the supernatant was collected. The beads were then resuspended in 100 µl of Elution Buffer II (2M UREA/50 mM Tris-HCl pH 7.5/5 mM IAA), incubated for 5 minutes, separated again on a magnetic rack, and the resulting supernatant was combined with the first elution. A total of 200 µl of eluate was pooled and incubated overnight at room temperature. The digests were then acidified with 1 µl of trifluoroacetic acid (TFA), purified on C18 StageTips (Pierce), and vacuum dried. The dried peptides were resuspended in 2% acetonitrile/0.1% TFA prior to LC-MS/MS analysis.

Liquid chromatography – mass spectrometry (LC-MS) Analysis: Peptides were analyzed by mass spectrometry at the Wohl Institute for Translational Medicine Proteomics Unit, Sheba Medical Center, Israel. Purified peptides were separated using the EASYnLC1200 nano-HPLC system with EasySpray columns (PepMap, 50 cm length, 0.75 µm inner diameter) packed with 2 µm C18 material with a 100 Å pore size. Separation was performed using a water-acetonitrile gradient, and peptides were injected into the Q-Exactive HF mass spectrometer (Thermo Scientific) via the EasySpray ionization source. The peptides were loaded with Buffer A (0.1% formic acid) and eluted with a gradient of 5–30% Buffer B (80% acetonitrile/0.1% formic acid) at a flow rate of 300 nl/min over 100 minutes.

Mass Spectrometric Acquisition: MS acquisition was performed in data-independent acquisition (DIA) mode under positive-ion conditions. The DIA method included a survey scan at 60,000

resolution from 400 to 1,000 m/z, with an automatic gain control (AGC) target of 3E6 and an injection time of 100 ms. Twenty-five DIA windows, spanning 400–975 m/z, were acquired at 30,000 resolution with an AGC target of 1E6 and a 50 ms injection time. Normalized collision energy was set to 27.

Mass Spectrometric Raw Data Analysis: The raw MS files were processed using DIA-NN 1.8.1 with global cross-run normalization enabled. The output was filtered at a 1% false discovery rate (FDR) at both the precursor and protein group levels. Protein quantification was performed using the MaxLFQ algorithm as implemented in the `diann` R package. Database searching was conducted against the Human UniProt database in library-free mode.

Statistical analysis

To assess differences in kidney failure age, consanguinity rate, and the percentage of cases with a family history between genetically solved cases and those without a genetic etiology, we conducted an unpaired, two-tailed t-test. To examine the association between terminal complement variants and kidney disease, we compared the minor allele frequency (MAF) in our cohort with that of 399 ethnically matched controls without kidney disease. To ensure no selection bias was evident in cases or controls, we first confirmed that both cases and control cohorts did not deviate from Hardy-Weinberg equilibrium (P = 0.6 and P = 1) based on allele frequencies in the control cohort and accounting for an inbreeding coefficient of 0.0238[S4,S20]. This was assessed using the Freeman-Halton extension of Fisher's exact test (two-tailed probability). Subsequently, we assessed the association between the risk genotype and kidney failure by comparing the observed and expected genotype frequencies in cases and controls using the Freeman-Halton extension of Fisher's exact test. Minor allele frequencies as well as homozygotes and carrier rates were compared between cases and controls using two-sided Z test for two proportions.

Table S1

Table S1. Clinical characteristics and pre-exome sequencing diagnosis of 81 Bedouin persons with kidney failure.		
**Characteristics**	**Value**	**Percentage**
**Gender**		
Male	47	58%
Female	34	42%
**Phenotype**		
Nephropathy of unknown cause	23	28%
Diabetic nephropathy	22	27%
Glomerulopathy	19	23%
CAKUT	8	10%
Hypertensive nephropathy	4	5%
Polycystic kidney disease	2	2%
Other	3	4%
**Biopsied**		
Yes	18	22%
No	63	78%
**Age at kidney failure (years)**		
0-19	14	17%
20-39	19	23%
40-59	28	35%
>60	20	25%
**Parental consanguinity**		
First cousins	16	20%
More distant relatives	10	12%

Unrelated	55	68%
**Family members with kidney failure**		
Yes	24	30%
No	57	70%
Patients were classified as having a family history of kidney failure if they had at least one second-degree relative or closer who underwent chronic dialysis or kidney transplantation at any age. CAKUT: congenital anomalies of the kidney and urinary tract.		

Table S2

Table S2. Effect of post-ES molecular-genetic diagnoses on clinical management.									
**Individual**	**Pre-ES clinical diagnosis**	**Gene**	**Zygosity**	**Clinical management implications**					**FHx**
				**Screening of family members**	**Genetic counselling for family planning**	**Consideration during living donor selection**	**Surveillance of extra-renal phenotypes in affected individuals**	**Other**	
**Monogenic kidney diseases**									
**21-759**	Male with hearing loss, familial hematuria, normal sized kidneys with multiple cortical kidney cysts; clinical diagnosis of ADPKD; kidney failure at 54 Y/O. Currently transplanted.	***COL4A3***	Hom	+	+		ENT surveillance and re-evaluation of hearing problems. Ophthalmologic evaluation to identify ophthalmic diseases such as anterior lenticonus.	May allow patient to become a candidate for novel future treatments[S21].	Yes
**21-1209**	Male with long standing microscopic hematuria, obesity, DM; clinical diagnosis of diabetic nephropathy; kidney failure at 64 Y/O. Currently on chronic dialysis.	***COL4A3***	Het	+	+	+		May allow patient to become a candidate for novel future treatments[S21].	Yes
**21-990**	Male with longstanding microscopic hematuria; clinical diagnosis of nephropathy of unknown cause; kidney failure at 35 Y/O. Currently transplanted.	***COL4A3***	Het	+	+			May allow patient to become a candidate for novel future treatments[S21].	Yes
**21-1614**	Female with nephrolithiasis, proteinuria; clinical diagnosis of nephropathy of unknown cause; kidney failure at 54 Y/O. Currently transplanted.	***COL4A3***	Het	+	+			May allow patient to become a candidate for novel future treatments[S21].	No
**21-715**	Female with DM, clinical diagnosis of diabetic nephropathy; kidney failure at 73 Y/O. Currently on chronic dialysis.	***COL4A3***	Het	+	+	+		May allow patient to become a candidate for novel future treatments[S21].	No

**21-755**	Female with clinical and molecular diagnosis of GSD type 1b, hearing loss and intellectual disability; kidney failure at 20 Y/O; parental consanguinity. Currently transplanted.	***SLC37A4***	Hom		+		Surveillance for GSD 1 subtype-specific complications such as risk of neutropenia and inflammatory bowel disease.	The patient had a prior molecular diagnosis.	No
**21-1028**	Female with FSGS, CAKUT, hypothyroidism; kidney failure at 20 Y/O; Currently on chronic dialysis.	***PAX2***	Het	+	+	+	Ophthalmologic evaluation to identify ophthalmic disease such as optic nerve or retinal coloboma.		No
**21-1064**	Male with heart failure and clinical diagnosis of nephropathy of unknown cause; kidney failure at 20 Y/O; parental consanguinity. Currently on chronic dialysis.	***NPHP4***	Hom		+	+	Ophthalmologic evaluation for ocular involvement such as ocular motor apraxia and retinitis pigmentosa.		No
**21-1210**	Female with recurrent arterial and venous thrombosis, HUS with no diarrheal prodrome; kidney failure at 51 Y/O. Currently on chronic dialysis.	***CFH***	Het	+	+	+		Informs the use of post-transplant Eculizumab (this specific patient was not a transplant candidate due to vascular complications).	Yes
**21-1311**	Male with clinical diagnosis of ADPKD; kidney failure at 48 Y/O. Currently on chronic dialysis.	***PKD2***	Het	+	+	+	Consider need for brain imaging to screen for intracranial aneurysms and echocardiography to assess for valvular disorders based on specific risk factors.	May allow patient to become a candidate for novel future treatments[S22].	Yes
**Other monogenic diseases**									
**21-179**	Male with IgA nephropathy; kidney failure at 13 Y/O; parental consanguinity. Currently transplanted.	***C8A***	Hom	+	+			Antibiotic prophylaxis for affected individuals	No
**21-1016**	Male with IgA nephropathy; kidney failure at 13 Y/O; parental consanguinity. Currently transplanted.	***C8A***	Hom	+	+			Antibiotic prophylaxis for affected individuals	No

**21-753**	Male with an early onset DM, diabetic nephropathy; kidney failure at 32 Y/O; parental consanguinity. Currently transplanted.	***C8B***	Hom	+	+			Antibiotic prophylaxis for affected individuals	No

Patient’s phenotype and affected gene are presented alongside the clinical implications of the molecular diagnosis. Abbreviations: ADPKD, autosomal dominant polycystic kidney disease; CAKUT, congenital anomalies of the kidney and urinary tract; DM, diabetes mellitus; ES, exome sequencing; FHx, family history of kidney disease; FSGS, focal segmental glomerulosclerosis; GSD, glycogen storage disease; Het, heterozygous; Hom, homozygous; HUS, hemolytic uremic syndrome

Table S3

Table S3: Total serum complement activity (CH50), complement components and immunoglobulins level in patients with kidney failure and pathogenic variants in terminal complement genes.				
**Patient Index**	**21-179**	**21-1016**	**21-753**	**21-922**
**Affected gene**	***C8A***	***C8A***	***C8B***	***C9***
**Complement activity**				
CH50 [U Eq/ml] (normal 144-240, low 9-50)	1.04	5.50	24.85	24.56
**Serum complement components**				
C3 [mg/dl] (90-180)	77.00	94.20	111.00	119.00
C4 [mg/dl] (10-40)	21.00	22.70	38.10	32.40
C6 [mg/L] (70-86)	75.09	108.92	124.68	93.35
C7 [mg/L] (60-80)	229.98	115.12	171.99	69.26
C8 [mg/L] (105-127)	0.00	0.00	88.33	113.29
C9 [mg/L] (270-330)	279.80	350.48	637.23	6.20
**Serum immunoglobulin levels**				
IgG [mg/dl] (670-1530)	377.00	1050.00	1090.00	1770.00
IgA [mg/dl] (52-274)	111.00	279.00	235.00	158.00
IgM [mg/dl] (62-231)	37.00	156.00	68.10	167.00
Reference values are presented in brackets. Clinically significant abnormal values appear with gray background.				

Figure S1

Diagnostic yield of exome sequencing in the Israeli Bedouin kidney failure population. a: Monogenic kidney disease was diagnosed in 12% of cases. Of those, the most common affected gene was *COL4A3*. b: The pre-exome clinical diagnosis, as opposed to the molecular genetic (post-exome) diagnosis.

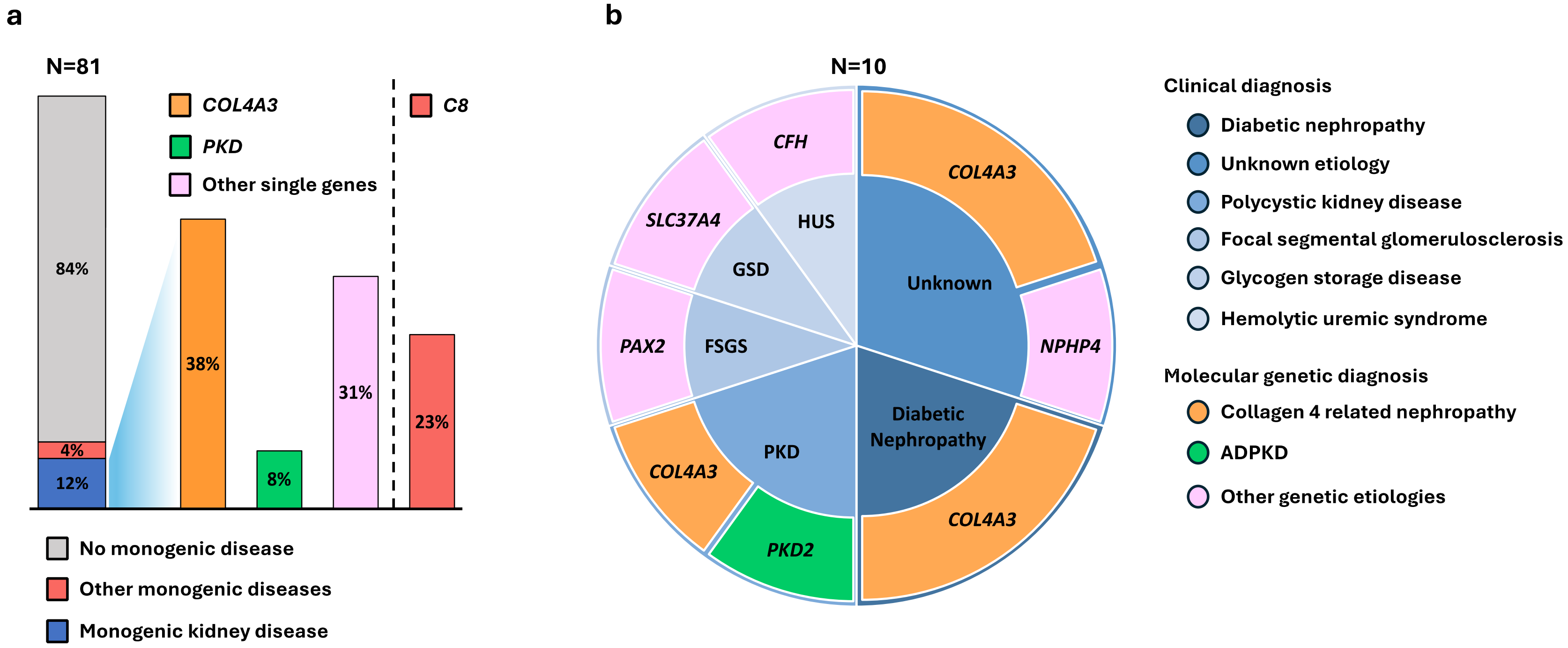

Figure S2

Histopathology of kidney biopsy obtained from 179-21 (*C8A* truncating variant) showing IgA Nephropathy. The biopsy was taken at the age of 13 years from the patient's native kidney. Both light microscopy and immunofluorescence are presented. H&E: Hematoxylin and Eosin stain, PAS: Periodic acid Schiff stain, JMS: Jones' Methenamine Silver stain.

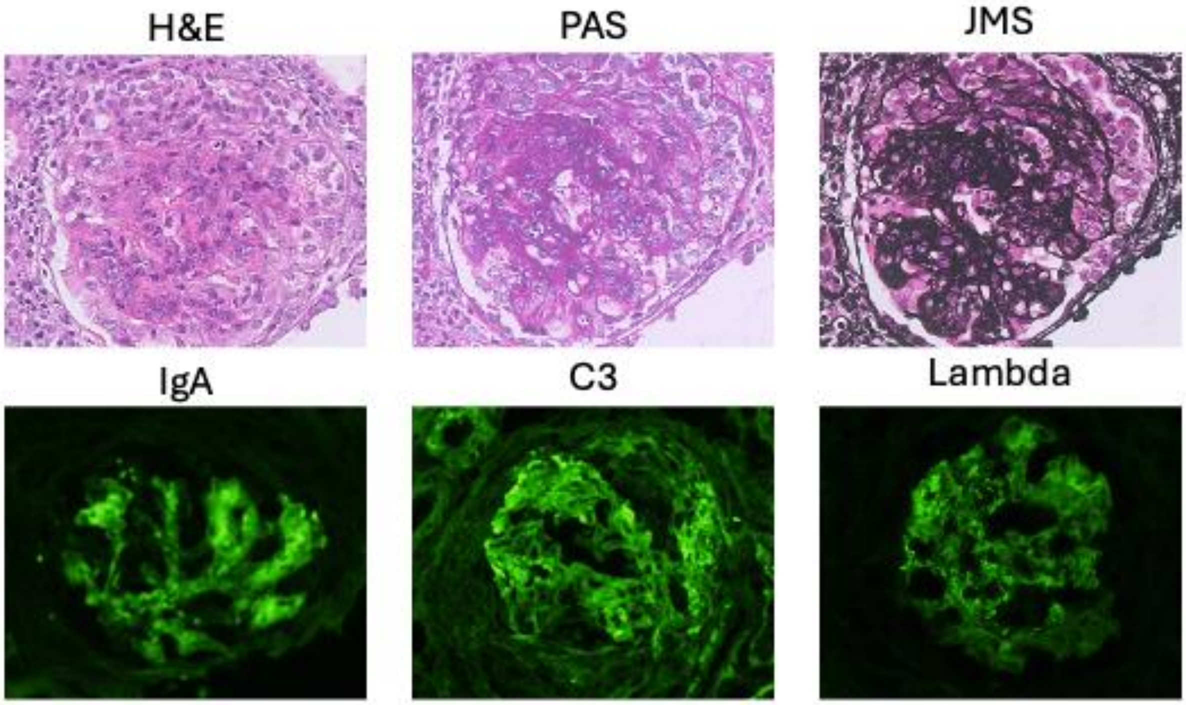

Supplementary references

S1. The National Registry of Renal Replacement Therapy (Dialysis and Renal Transplants) - Renal Replacement Therapy in Israel 1990-2015.

S2. Regev-Epstein LC, Frishberg Y, Davidovits M, et al. Dialysis in Israeli Children between 1990 and 2020: Trends and International Comparisons. *Clinical Journal of the American Society of Nephrology*. 2023;18(3):363-373. doi:10.2215/cjn.0000000000000063

S3. Markus B, Alshafee I, Birk OS. Deciphering the fine-structure of tribal admixture in the Bedouin population using genomic data. *Heredity (Edinb)*. Feb 2014;112(2):182-9. doi:10.1038/hdy.2013.90

S4. Na'amnih W, Romano-Zelekha O, Kabaha A, et al. Prevalence of consanguineous marriages and associated factors among Israeli Bedouins. *J Community Genet*. Oct 2014;5(4):395-8. doi:10.1007/s12687-014-0188-y

S5. Zeni L, Mescia F, Toso D, et al. Clinical Significance of the Cystic Phenotype in Alport Syndrome. *Am J Kidney Dis*. Sep 2024;84(3):320-328 e1. doi:10.1053/j.ajkd.2024.02.005

S6. Graziani L, Minotti C, Carriero ML, et al. A Novel COL4A5 Pathogenic Variant Joins the Dots in a Family with a Synchronous Diagnosis of Alport Syndrome and Polycystic Kidney Disease. *Genes (Basel)*. May 8 2024;15(5)doi:10.3390/geneS15050597

S7. Kircher M, Witten DM, Jain P, O'roak BJ, Cooper GM, Shendure J. A general framework for estimating the relative pathogenicity of human genetic variants. *Nature genetics*. 2014;46(3):310-315.

S8. Chen S, Francioli LC, Goodrich JK, et al. A genomic mutational constraint map using variation in 76,156 human genomes. Nature. Jan 2024;625(7993):92-100. doi:10.1038/s41586-023-06045-0

S9. Kaufeld J, Reinhardt M, Schroder C, et al. Atypical Hemolytic and Uremic Syndrome Triggered by Infection With SARS-CoV2. *Kidney Int Rep*. Oct 2021;6(10):2709-2712. doi:10.1016/j.ekir.2021.07.004

S10. Walker LC, Hoya M, Wiggins GAR, et al. Using the ACMG/AMP framework to capture evidence related to predicted and observed impact on splicing: Recommendations from the

ClinGen SVI Splicing Subgroup. *Am J Hum Genet*. 2023;110(7):1046-1067. doi:10.1016/j.ajhg.2023.06.002

S11. Lata S, Marasa M, Li Y, et al. Whole-Exome Sequencing in Adults With Chronic Kidney Disease: A Pilot Study. *Ann Intern Med*. Jan 16 2018;168(2):100-109. doi:10.7326/M17-1319

S12. Welch TR, Blystone LW. Renal disease associated with inherited disorders of the complement system. Pediatr Nephrol. Aug 2009;24(8):1439-44. doi:10.1007/s00467-008-1027-3

S13. Fu X, Ju J, Lin Z, et al. Target deletion of complement component 9 attenuates antibody-mediated hemolysis and lipopolysaccharide (LPS)-induced acute shock in mice. *Sci Rep*. Jul 22 2016;6:30239. doi:10.1038/srep30239

S14. Mohammed Y, Michaud SA, Petrosova H, et al. Proteotyping of knockout mouse strains reveals sex- and strain-specific signatures in blood plasma. *NPJ Syst Biol Appl*. May 28 2021;7(1):25. doi:10.1038/s41540-021-00184-8

S15. Schroder-Braunstein J, Kirschfink M. Complement deficiencies and dysregulation: Pathophysiological consequences, modern analysis, and clinical management. *Mol Immunol*. Oct 2019;114:299-311. doi:10.1016/j.molimm.2019.08.002

S16. Waterhouse A, Bertoni M, Bienert S, et al. SWISS-MODEL: homology modelling of protein structures and complexes. *Nucleic acids research*. 2018;46(W1):W296-W303.

S17. McKenna A, Hanna M, Banks E, et al. The Genome Analysis Toolkit: a MapReduce framework for analyzing next-generation DNA sequencing data. *Genome research*. 2010;20(9):1297-1303.

S18. Nykamp K, Anderson M, Powers M, et al. Sherloc: a comprehensive refinement of the ACMG-AMP variant classification criteria. *Genet Med*. Oct 2017;19(10):1105-1117. doi:10.1038/gim.2017.37

S19. Einhorn Y, Einhorn M, Kamshov A, et al. Gene-specific artificial intelligence-based variant classification engine: results of a time-capsule experiment. Research Square; 2019.

S20. Hartl DL, Clark AG. *Principles of population genetics*. 4th ed. Sinauer Associates; 2007:xv, 652 p.

S21. Reiterova J, Tesar V. Current and Future Therapeutical Options in Alport Syndrome. *Int J Mol Sci*. Mar 14 2023;24(6)doi:10.3390/ijms24065522

S22. Capelli I, Lerario S, Ciurli F, et al. Investigational agents for autosomal dominant polycystic kidney disease: preclinical and early phase study insights. Expert Opin Investig Drugs. May 2024;33(5):469-484. doi:10.1080/13543784.2024.2342327
